# Bridge hosts, a missing link for disease ecology in multi-host systems

**DOI:** 10.1186/s13567-015-0217-9

**Published:** 2015-07-21

**Authors:** Alexandre Caron, Julien Cappelle, Graeme S Cumming, Michel de Garine-Wichatitsky, Nicolas Gaidet

**Affiliations:** UR AGIRs, Cirad, Montpellier, France; UR AGIRs, Cirad-RP-PCP, Harare, Zimbabwe; Mammal Research Institute, University of Pretoria, Pretoria, South Africa; Epidemiology and Public Health Unit, Institut Pasteur du Cambodge, Phnom Penh, Cambodia; Percy FitzPatrick Institute, University of Cape Town, Cape Town, South Africa; Department of Biological Sciences, University of Zimbabwe, Harare, Zimbabwe

## Abstract

In ecology, the grouping of species into functional groups has played a valuable role in simplifying ecological complexity. In epidemiology, further clarifications of epidemiological functions are needed: while host roles may be defined, they are often used loosely, partly because of a lack of clarity on the relationships between a host’s function and its epidemiological role. Here we focus on the definition of bridge hosts and their epidemiological consequences. Bridge hosts provide a link through which pathogens can be transmitted from maintenance host populations or communities to receptive populations that people want to protect (i.e., target hosts). A bridge host should (1) be competent for the pathogen or able to mechanically transmit it; and (2) come into direct contact or share habitat with both maintenance and target populations. Demonstration of bridging requires an operational framework that integrates ecological and epidemiological approaches. We illustrate this framework using the example of the transmission of Avian Influenza Viruses across wild bird/poultry interfaces in Africa and discuss a range of other examples that demonstrate the usefulness of our definition for other multi-host systems. Bridge hosts can be particularly important for understanding and managing infectious disease dynamics in multi-host systems at wildlife/domestic/human interfaces, including emerging infections.

**Electronic supplementary material:**

The online version of this article (doi:10.1186/s13567-015-0217-9) contains supplementary material, which is available to authorized users.

**Table of contents**

1. A functional approach to disease ecology
2. Transmission function and bridge host
3. A framework to identify bridge hosts for AIV
4. Bridge hosts and other multi-host systems
5. Implication for disease ecology
6. Conclusions
7. Competing interests
8. Authors’ contributions
9. Acknowledgements
10. References

## A functional approach to disease ecology

Ecological functional approaches classify organisms according to what they do, and/or what they eat. They offer an alternative perspective to taxonomic classifications for identifying trends within and making sense of ecological complexity. Applications of functional group concepts, which date back to fundamental ideas about biomass distributions across different trophic levels [1], have been crucial in advancing ecological understanding. More recently, ecological functional analyses have achieved prominence as a way of linking taxonomic survey data and the provision of ecosystem services [2]. Functional analyses thus remain an important research area in ecology.

In epidemiology, functional concepts have clear potential utility but are still in a relatively early stage of development. Classical epidemiology relies heavily on single-species studies, particularly those of people (e.g., analyses of measles and smallpox in human populations [3]). In contemporary epidemiological studies, in the last fifteen years, under the influence of ecology, the scope of epidemiology is being broadened to include plant and animal communities in which multiple different species can contribute to the maintenance and spread of pathogens in host populations [4]. In multi-host systems, the role played by each host population in pathogen dynamics is determined by the species’ competence for the pathogen (i.e., its receptivity to infection and its capacity to replicate and transmit the pathogen [5]), its exposure to the pathogen determined by the host ecology/behaviour and its interactions with other host populations (including vectors for vector-borne infections) leading to infectious contacts, and finally, the composition of the host community that will determine the range of inter-host interactions [6].

One of the central questions in disease ecology is that of how the community composition of potential host species relates to the dynamics of pathogen transmission within the host community, as opposed to within a population of a single species. The complexity of this problem can be simplified by assigning epidemiological functions to relevant traits that define an organism’s role in the epidemiology of a given pathogen. For example, animals that undertake long movements (a trait) may contribute to the epidemiological function (pathogen disperser) of spreading pathogens over large distances (a role). Grouping organisms by epidemiological functions facilitates the development of eco-epidemiological models for a given pathogen in relation to an entire animal community [7]. This approach could potentially play an important role in guiding research, as well as in the surveillance and control of animal and zoonotic diseases [8].

Although some progress has been made in the characterization of epidemiological functional groups, (e.g., clear definition of the *maintenance function* [9,10]), other epidemiological functions remain incompletely defined, especially those relating to the transmission of pathogens between groups of hosts. In this paper we first define the *transmission function* in relation to the *maintenance function*. We then focus on the concept of “bridge hosts” and demonstrate their potential importance in the ecology of disease transmission in multi-host systems. Though closely related concepts have been used previously [10-12], we believe that a refined definition embedded in a clear functional framework is still lacking. Lastly, we present an operational framework to identify potential bridge host populations, using as a case study the ecology of avian influenza viruses at the wild/domestic bird interface in Africa and also giving other multi-host systems examples.

## Transmission function and bridge host

We use “host” to refer to a host population, a host species, or a host community. The smallest epidemiological unit to which we will refer is a host population, acknowledging the fact that individual variability can also substantially impact pathogen transmission (e.g. “superspreader”, [13]). As defined by Haydon et al. [10] and more recently revised by Viana et al. [9], a conceptual framework for the role of hosts in epidemiology requires the definition of the *target* host: “the population of concern to the observer” in the geographic area under study (Table 1). A maintenance host will only be relevant to a target population if it can be in contact with and able to transmit the infection to it.

**Table 1:** **Role of hosts in pathogen epidemiology and their participation in maintenance and transmission functions**

**Role of hosts in pathogen epidemiology**	**Definition or related definition for the case of bridge host**	**Maintenance Function**	**Transmission Function**	**Examples**
**Target host**	- The population of concern to the observer [10]	X		- Human populations (for zoonoses)
				- Domestic populations
				- Threatened wildlife species
**Maintenance host population**	- Hosts in which the pathogen persists even in the complete absence of transmission from other hosts [12]	X	(X)	- Brush-tailed possums for bovine tuberculosis in New Zealand [12]
	- Population larger than the critical community size (i.e. size under which the pathogen cannot be maintained in the community) in which the pathogen persists [10]			- White-footed mouse (*Peromyscus leucopus*) for Lyme disease in the United States [6]
**Maintenance host community/Maintenance host complex**	- One or more epidemiologically connected populations or environments in which the pathogen can be permanently maintained [10]	X	(X)	- Anatids for avian influenza viruses worldwide [24]
	- Any host complex in which disease persists indefinitely is a reservoir [12]			- Amphibian sp. for the trematode *Ribeiroia ondatrae* [18]
	- Host for which cross species transmission and inter-species transmission are high [14]			
**Bridge host**	- Non-maintenance host population able to transmit a pathogen from a maintenance host/complex to the target population, otherwise not or loosely connected to the maintenance complex **(this manuscript)**		X	
	Previous related definitions:			- Little studied so far
	- *Source population*: any population that transmits infection directly to the target population [10]			- Red deer and domestic pigs for bovine tuberculosis in New Zealand [12]?
	- *Liaison host*: incidental hosts that transmit pathogens from a reservoir to another incidental host [11,15]			- Peri-domestic birds such as swallow sp., sparrow sp., etc. [23]
	- *Spatial vector*: host that transport the pathogen to target populations in new locations [12]			
	- *Temporal vector*: host that can transmit the pathogen to target species across temporal scale [12]			

Crosses in brackets indicate that maintenance host can participate in the transmission function although this is not a necessary condition.

The *maintenance function* represents the capacity to maintain the pathogen within the ecosystem. A *maintenance host* is a host population (single population) or community/host complex (several sympatric host populations) “in which the pathogen persists even in the complete absence of transmission from other hosts” [12]. The maintenance function depends on host density, and on intra- and inter-host infectious contacts (i.e., a contact leading to infection amongst other intra-host factors; [14]). In multi-host systems, the notion of a maintenance community in which several populations from different species play a role in the maintenance of the pathogen seems more appropriate than the “reservoir” concept [11,15] for understanding pathogen dynamics. The reservoir concept is still being used in contradictory ways, as discussed by several authors [10-12]. Haydon et al. [10] extended the definition of reservoir by adding “source populations” that may not be involved in the maintenance of the pathogen but rather in the transmission of the pathogen to the target population. Ashford [11] defined a “liaison host” as linking the reservoir to another host population, with no explicit reference to target populations. We agree with Ashford [11] that source population should not be included in the definition of the reservoir, as this term is strongly linked to the concept of maintenance and because control of infection in the reservoir would be different if targeted at the maintenance or source populations. For example, aiming at controlling the infection in a maintenance vs. a source population might have different outcomes, since the maintenance host could still re-infect the source population in the latter case. To add to the confusion, Suzán et al. [16] presented a new framework to understand patterns in space and time of meta-communities of hosts and parasites. In their first figure they display in red “reservoir species” and in orange “alternative hosts”, together “maintaining higher infection of prevalence”. Clearly, their concept of “reservoir” differs from that of Haydon et al. [10], who argued that any host involved in the maintenance of the pathogen should be part of the reservoir. The difference in definitions is identical with Plowright et al. [17]: they present domestic horses as potential source populations (defined in the article as “recipient” and “intermediate hosts”) of Hendra viruses for human populations without considering them as part of the reservoir (presented as the bat community). The extensive use of the “reservoir” concept under multiple definitions and the lack of consensus around the liaison host and source population concepts (revealed by the scarcity of use of these two last terms in the literature) requires a refined conceptual framework and definitions. Agreeing with others [11,12], we thus prefer to use only maintenance host or community, a term that refers better to the dynamic aspect of the functional role than the static notion of a reservoir [6,18].

Although the maintenance-target host relationship and its link with the maintenance function have been properly defined, the function of pathogen transmission to the target host needs a clearer definition. Interspecific pathogen transmission is of crucial importance for infectious disease management. Disease control can target the maintenance host to stop pathogen maintenance and circulation in the ecosystem (i.e. targeting the maintenance function); however, as this option is often unfeasible (for practical or ethical reasons, notably concerning wildlife populations), one could also try to break the transmission pathway that brings the pathogen to the target host. We therefore define the *transmission function* as the capacity to transmit the pathogen to the target host. This function must be separated from the maintenance function, as the maintenance host does not always have infectious contact with the target host. When it has direct contact with the target host, then the maintenance host is implicated in the maintenance and transmission functions. When it does not, a *bridge host* (Table 1) can connect (i.e., have infectious contact with) both maintenance and target hosts, “bridging” the gap between them. Using this functional definition, the concept of the reservoir as revisited by Haydon et al. [10] and more recently by Viana et al. [9], does not refer clearly to a single epidemiological function, because it includes maintenance host(s) involved in the maintenance function and potentially in the transmission function as well as non-maintenance population(s) only involved in the transmission function. Allocating hosts belonging to the reservoir to specific functional groups that surveillance and/or control can target is therefore difficult and provides an additional reason to focus solely on the maintenance-target hosts.

*Bridge host* is therefore used, since (i) the group is distinct from the source population, as bridge hosts do not belong to the maintenance host/community, and the liaison host as a bridge host is always in reference to a maintenance-target population system; and (ii) the word “bridge” is relevant to the definition proposed (e.g. [19]). Bridge hosts refer therefore to a group of hosts that perform the same epidemiological function for a pathogen that can be targeted by specific surveillance and control interventions. In Suzán et al. [16], information about whether alternative hosts function as bridge hosts would add an important layer of information to their framework and contribute to the understanding of the spatial spread of parasites.

Our bridge host definition is closely related to the “spatial and temporal vector” concepts presented by Nugent [12] but unifies them with previous definitions (see above) and broadens them. A bridge host can be defined at the level of a population or a community. Bridge hosts may be frequent in disease ecology, but this term has not been explicitly defined and its usage is not common when referring to the transmission function without any role in maintenance function. For example, it would be incorrect to use the term “bridge species” as the role of a bridge host would refer to a specific host population in interaction with other hosts in a given ecosystem (e.g. contact with maintenance or target populations) and at a specific density [12]; the host density and the network of interaction between these hosts in another ecosystem would likely be different and would make it unlikely that a species can play the same epidemiological functional role across its range.

A clearer conceptual framework is thus needed to guide the identification of bridge hosts and the characterisation of their roles in disease ecology. This framework must also be operationalised if it is to guide the design of hypotheses that can be tested through field protocols to characterise the role(s) of hosts in disease ecology.

**Figure 1:**
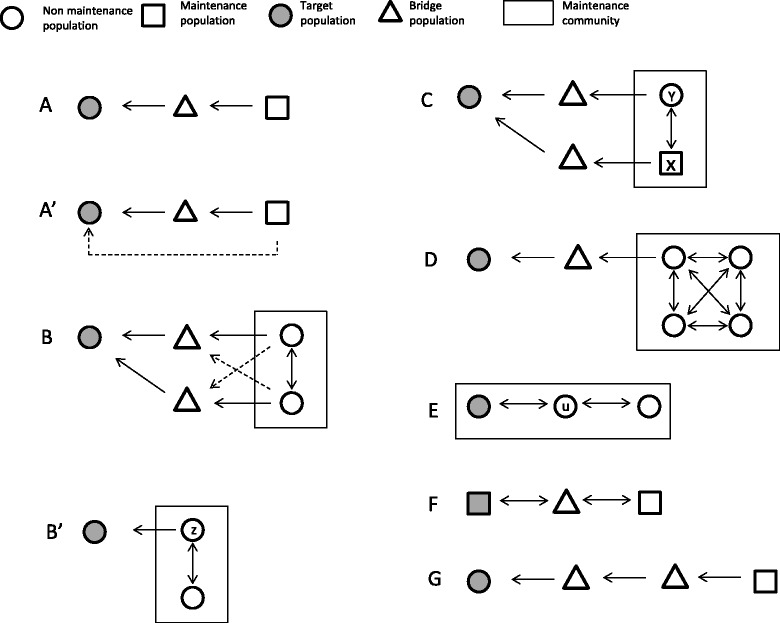
**Definition of different target-bridge-maintenance systems (adapted from Haydon et al. [** 10 **]).** A represents the simplest maintenance-bridge-target system. In A’, the maintenance and target populations are less connected (frequency/intensity of infectious contacts) than between the maintenance-bridge-target populations. In B, mitigation strategies aimed at one bridge host cannot fully control pathogen transmission to the target host because of the alternative bridge host’s pathway. If both maintenance populations were in contact with both bridge hosts (i.e. if dashed arrows exist), controlling contacts between the target population and bridge hosts should be simpler than other control options. In B’, according to our definition, Z is not considered as a bridge population as it belongs to the maintenance community. In C, stopping contacts between the maintenance population and the target population by acting on one of the two bridge hosts would not be enough to stop transmission, which can still occur through the second bridge host. D is a special case of B, understanding the complexity of the maintenance community is not necessary to control the pathogen transmission risk to the target population, which can be achieved through the control of arrows connecting the bridge host. In E, none of the host populations can sustain the infection by itself and according to our definition, u is not considered as a bridge population as it belongs to the maintenance community. In F, the bridge host connects the target population with another maintenance host creating a system with a maintenance meta-population, which could change the epidemiological dynamics of the system and the probability of success of intervention strategies (e.g. vaccination coverage to achieve control of the infection in the target population). G is a special case where two bridge hosts are necessary to achieve the transmission function. Good knowledge of the ecological interactions in the ecosystem will be necessary to identify such complex interactions between bridge hosts.

Using the different target-maintenance systems proposed by Haydon et al. [10], bridge hosts can be included in target-bridge-maintenance systems in several ways (Figure 1). According to our definition, a bridge host is involved in the transmission function while not involved in the maintenance function. Two main prerequisites must be fulfilled for a host to qualify as a bridge host. The first prerequisite is that the host must be competent for the pathogen (i.e., must be receptive to infection, permit pathogen replication, and be able to excrete it) without being able to maintain it alone, in which case the host would be considered as a maintenance host; or alternatively, the host should be able to mechanically transport the pathogen [20,21]. Its competence will influence the capacity of a bridge host to achieve the transmission function: if the bridge host has a short pathogen excretion period, it will be able to transmit the pathogen to a target population only if the time lag between contact with a maintenance and then a target host is shorter than the excretion period, or if the distance between target and maintenance is shorter than the maximum distance that the bridge host can travel during its excretion phase. Similarly, for mechanical transmission, the survival of the pathogen on/in the host body part (e.g. skin, hair, mouth, feathers) exposed to the external environment will determine for how long the host can play the bridge role.

The second prerequisite is that infectious contacts must occur along the maintenance-bridge-target transmission chain. These will depend on direct and indirect (e.g. environmental transmission) contacts, the mode of transmission of the pathogen, and the site of infection. The basic reproductive number R_0_ for the bridge host (not considering here mechanical transmission) should be <1 as it cannot maintain the infection but its force of infection, dependent on the number and extent of infectious contacts with the target host, can be high.

A bridge host that compensates for a lack of infectious contacts between maintenance and target hosts can operate across different dimensions: spatial, temporal, and behavioural. The *spatial dimension* arises when the bridge host creates a spatial link between the separate areas in which the maintenance and the target host populations occur. This dimension typically refers to the situations developed below for wild birds and avian influenza. It has been defined as a “spatial vector” by Nugent [12] when considering the role of feral pigs (*Sus scrofa*) in the epidemiology of bovine tuberculosis (bTB) in New Zealand.

The *temporal dimension* arises when the pathogen can persist (but not be maintained indefinitely) in the bridge host for a period of time longer than in the maintenance host or during a distinct season; this has been well described by Nugent [12] as a “temporal vector”, for example when red deer (*Cervus elaphus*) transmit *Mycobacterium bovis* to possum populations that are controlled to levels that are well under the critical community size for bTB maintenance.

The *behavioural dimension* exists when the absence of contact between sympatric maintenance and target hosts is compensated for by another host that has infectious contacts with both. Situations may occur in which the microhabitat preferences and behaviours of maintenance and target hosts mean that they do not come into direct contact despite using the same locations on a daily basis. Bats, for example, are believed to be the maintenance host for Ebola, and can be sympatric with people; but Ebola transmission from bats to humans is enhanced by the great apes (whose susceptibility to Ebola seems to indicate that they are not maintenance hosts) which feed with bats and are fed upon by humans [22]. It is interesting to note that in all cases, even a R_0_ close to zero (approximating a dead-end host) could still be important for the transmission function: the capacity to excrete the pathogen for a few hours, associated with some form of dispersal, may be sufficient for a bridge host to come into contact with the target host and infect it. For pathogens like Ebola, the range of hosts that is classically considered to be important in disease ecology may have to be broadened by including hosts that are able to transmit the pathogen over short time- and space-scales. These hosts are commonly considered as playing no role in pathogen ecology and are called dead-end hosts (e.g., most wild avian hosts for avian influenza virus - AIV - apart from Anseriformes and Charadriiformes). Amongst the multitude of those dead-end hosts, the bridge host perspective can identify some that do play a role in disease ecology.

With this framework in place, we next turn to the question of how bridge hosts can be identified in the multi-host context of AIV epidemiology and suggest an operational framework (partially implemented in [23]) that can enhance disease ecology as well as pathogen surveillance and control.

## A framework to identify bridge hosts for AIV

Waterfowl (defined here as ducks, geese, waders, gulls, and terns) constitute the maintenance hosts for low pathogenic avian influenza viruses (LPAIV) [24]. AIV represent major threats to poultry production when strains originating from wild birds evolve from low to high pathogenicity in the poultry (target) populations [25]. The transmission of LPAIV between the wild bird maintenance community and domestic populations is therefore crucial to managing the sanitary and economic impacts of the disease. In this section, the risk of LPAIV spillover to poultry populations from the maintenance populations will be used as an example.

When poultry are confined in farms or buildings, their direct contacts with the maintenance waterfowl community, which mainly lives in wetlands and on coastal shorelines, are believed to be limited due to spatial segregation between populations. Many outbreaks of highly pathogenic AIV outbreaks have nonetheless occurred in domestic poultry production systems. It is therefore suspected that bridge hosts play a role in transmitting waterfowl-derived strains of AIV to poultry populations.

The ability of wild birds to travel long distances, and their ubiquity in most habitats, facilitate the potential for wild bird species to act as bridge hosts. Several constraints limit a better understanding of AIV ecology in bird communities: 1) high host diversity, that can include several hundred species in a given ecosystem; 2) the costs of diagnostic techniques that limit the number and type of samples (e.g. cloacal/tracheal swabs, blood) that can be analysed; and 3) the impossibility of randomly sampling from bird communities because of bias in capture techniques (e.g. walk-in traps, mist-nets). As a consequence, the information available on most wild bird species is scarce and has been obtained mostly from by-catch (i.e. captured non-targeted species) of studies investigating AIV in maintenance waterfowl, resulting in small sample sizes that are inadequate to provide epidemiological understanding of the host roles in AIV ecology in Africa [26]. The following framework used in a recent study [23] and here developed in detail, aims at first gathering/collecting available ecological and epidemiological information; second, at synthesizing this information to provide a priority list of species that act as potential bridge hosts; and finally, at undertaking targeted sampling that can determine the competence of the high priority species and revisit the framed hypotheses.

The range of methods available to characterize host competence for AIV and contact patterns between maintenance, potential bridge and target host populations is drawn from the fields of epidemiology and avian ecology (Table 2). None of these methods alone is sufficient to identify a bridge host in a given ecosystem [9]. Molecular epidemiology (e.g. gene sequencing after virus isolation) could in principle be used to identify bridge species but it is very unlikely that related strains from three different host populations (i.e., maintenance, bridge and target hosts) are concurrently isolated except perhaps during a localised AIV outbreak. Virological surveillance (e.g. polymerase chain reaction - PCR techniques) can provide information about host contacts between potential bridge and maintenance hosts if data are collected close to wetlands where waterfowl communities are known to occur. Serological investigation (e.g. ELISA tests) can be cheaper than virological testing but provide less information on the timing of the infection [27,28]. However, a combination of epidemiological and ecological methods could provide the necessary information to infer the bridge role of a given host population.

**Table 2:** **Evaluation of available epidemiological and ecological methods to identify bridge species for AIV according to their contributions to informing about host competence or contacts, as well as their relative costs (i.e. time and resources)**

	**Host competence**			**Host contacts**		**Resources**	**Examples for AIV**
**Method**	**Receptivity**	**Replication**	**Excretion**	**Contact/Maintenance**	**Contact/Target**		
Experimental Infection	xxx	xxx	xxx			xxx	[32-35]
Risk Analysis				x	x	x	[41-43]
Serological investigation	x	x		x		xx	[27,28,30]
Virological investigation	xx	xx	xx	xx		xx	[19,23,24,26,29,31,37]
Telemetry study				xxx	xxx	xxx	[39]
Bird ringing and monitoring				xx	x	x	[40]
Bird counts				xx	xx	x	[8,23,26,41]
Molecular epidemiology	xx	xx	xx	xx	xx	xxx	[48,49]

As the number of crosses increases in the first 2 columns the methods provide better ecological or epidemiological information; in the last columns, cost increases as the number of crosses increases.

Taking into account these constraints, the proposed framework aims, first, to narrow the large number of species by ranking the most probable potential bridge hosts based on proxies of host competence and/or contacts between maintenance, target and potential bridge hosts. This step can be achieved using (or combining) available published field (e.g. [24,29-31]) and experimental epidemiological studies (e.g. [32,33]). However, most AIV experimental studies have concentrated so far on a very limited set of species (e.g. for LPAI [32,33] and for HPAI [34,35]). Reviewing available PCR viral data within a given area or region can provide information on the range of host species with a competence for AIV. For example, in sub-Saharan Africa, the available databases are poor representations of existing avian diversity (only 10.9% of all African species have been sampled, Additional files 1 and 2, and only a few species were tested with a sample size that would be sufficient to detect 1% AIV prevalence). This exercise can help with ranking the species or groups of species based on the rate of infection, which provides an initial prioritization list for future investigation (Additional file 1). However, one shortcoming of PCR data is to link detection of genetic material and state of infectiousness of the sampled individual [36], an issue that is often overlooked but particularly important for the identification of bridge hosts.

The first step of the proposed framework must also incorporate ecological data that provide information about the presence/abundance of potential bridge hosts in the ecosystem and their potential contacts with maintenance and target hosts. However, it is a challenge to provide evidence that contacts (1) occur; and (2) result in successful virus transmission. Different types of data can be used or collected, each with its own strengths and weaknesses: life history traits (e.g., abundance, gregarism, foraging and migratory behaviour) obtained from the literature can be used as risk factors for contacts between wild and domestic birds or exposure to infection [37,38]; contacts between wild and domestic birds can be estimated using satellite telemetry [39]; capture-recapture techniques indicate population size (e.g. using colour rings at a local scale) [40]; and observations at focal points that are at wild/domestic bird interfaces (e.g. around poultry production building) can be used to quantify interactions [41].

The second step is to synthesize the ecological and/or epidemiological data to rank bird species according to the likelihood that they play a bridge role in the ecosystem under study. Risk analysis can provide such a tool [38,41-43] and may be particularly important when no information is available for an ecosystem, or prior to a field survey, by highlighting the populations that could be targeted preferentially. Once the bridging potential of different species has been evaluated, the third step of the framework consists in testing the host competence of the most likely bridge hosts in the ecosystem through targeted sampling. For example, Caron et al. [23] applied this framework in a southern African ecosystem and identified bridge hosts by combining bird counts with selected sampling and PCR testing.

Targeted sampling facilitates the concentration of resources to obtain adequate sample sizes and relevant epidemiological information and comes in place of the practice of blind sampling from wild bird communities, which is usually biased by capture techniques. Hypotheses can be revisited iteratively as more is learned about the potential of highly ranked species to act as bridge hosts. This approach can also lead to the detection of inconsistencies in the initial model (e.g., the definition of the maintenance community) and the necessity to revisit it [8].

## Bridge hosts and other multi-host systems

Avian influenza provides a good example of a case in which paying conceptual and practical attention to bridge hosts can enhance our understanding of pathogen dynamics in multi-host systems. Although the use of the bridge host concept may not be relevant for all multi-host systems, it has the potential to contribute to structuring investigations on the ecology of emerging pathogens shared at wildlife/livestock interfaces. To illustrate this point we present two additional examples of multi-host systems. In the first, Ebola in West Africa, understanding could be improved by the use of the conceptual framework developed here. In the second and better-known system, bovine tuberculosis in New Zealand, bridge hosts have been identified and are an important component of the problem.

Ebola virus spilled over in early 2014 in West Africa from an unknown animal to the human index case. Knowledge of Ebola ecology is still limited, despite the first outbreak having being reported in 1976. Current understanding points at bats (Mammalia: Chiroptera) as potential maintenance hosts, and contact between humans and some bat species occurs through the bushmeat industry [44] as well as via bat droppings and occasional cases of sick bats that are handled by humans [17,45]. However, embracing the functional approach presented here makes sense to look for potential bridge hosts that could link maintenance bats and humans. A priori, scavenging pigs, dogs, other non-maintenance bat species and wild antelope can have direct or indirect (e.g. consumption/hunting) contact with humans [46]. Targeted surveillance of such species will provide information on their competence for the virus; and host interaction protocols that identify contact networks with maintenance and target populations can provide information on the potential for viral spread (e.g. [47]). Once the multi-host system is better understood (case B, C or D in Figure 1), it may be simpler to try to block transmission pathways from bridge hosts to human populations (e.g. through changes in behavior related to bridge host consumption by people) than to control the pathogen in the maintenance hosts. A similar yet less complex example was recently developed [17], indicating that domestic horses could be “bridge hosts” for Hendra viruses between bats (maintenance host) and humans (target host) even if it is not yet known if horses could maintain the virus or just act as a bridge between bats and humans [17].

As a second example of the utility of the bridge host framework, Nugent [12] offers a comprehensive description of the bTB multi-host system in New Zealand. The cattle industry in New Zealand suffers from continuous spillover of the bTB mycobacterium from the maintenance host, the brush-tailed possum (*Trichosurus vulpecula*). The control of possum populations by depopulation is mainly implemented in areas around farms that are at high-risk of transmission to cattle, leaving high densities of possums in more distant forest and providing a gradient of bTB prevalence. This apparently efficient strategy is, however, thwarted by three potential bridge hosts (feral pigs *Sus scrofa*, red deer *Cervus elaphus*, and feral ferrets *Mustela furo*) that are involved in transmission (case G in Figure 1, called “link-host” in the article but lacking a more conceptual definition). Infected pigs and deer with large home ranges may leave the forest to die (or be hunted) around farms, providing an opportunity for ferrets to become infected when feeding on carcasses and subsequently infecting cattle or possums. This study is particularly interesting for 3 reasons: (1) the complexity and low probability of the chain of events leading to infection of the target population do not prevent bTB occurrence and the failure of disease control; (2) disease control targeted at the maintenance population prevents the transmission link between the maintenance and target hosts but the transmission pathways built by bridge hosts (case A’ in Figure 1) reduce the effectiveness of control, proving the importance of considering this epidemiological function and host role; and (3) the plasticity of the roles of host populations in disease epidemiology, which is heavily influenced by the environmental, ecological and anthropological context.

## Implications for disease ecology

The concepts of transmission function and bridge host contribute to a better understanding of disease ecology in multi-host systems by clarifying the epidemiological processes that are relevant for disease transmission and maintenance. This perspective fits better with the way that people operationalize the complexity theory and makes it easier to develop models of these systems. When maintenance and target hosts are not in direct contact, pathogen transmission relies on successive infectious contacts along the chain of maintenance, bridge and target hosts. Bridge hosts can play a pertinent and legitimate role in disease ecology and could become the targets for surveillance and control for some multi-host systems. For example, in some ecosystems, domestic bird populations are rarely in direct contact with wild waterfowl populations but phylogenetic analyses have indicated that most precursors of HPAIV in gallinaceous poultry have originated from wild waterfowl [48], suggesting that bridge hosts play a role in AIV transmission at the wild/domestic bird interface. More recently, evidence supporting a role for some passerines (finches, sparrows) in the transmission of the avian-origin human influenza A (H7N9) to human and poultry in China [49] suggests a potential role for passerines as bridge hosts between poultry and humans.

The functional approach emphasizes the need to focus on transmission pathways between hosts (and their directionality) instead of relying solely on intrinsic host properties (e.g. density, shedding capacity) [50,51]. The presence of a target host defines directionality in the transmission processes and implies a network of inter-connected hosts with different epidemiological roles. Our framework thus provides a better empirical approach to some kinds of epidemiological problems, such as the risk of spread of a specific pathogen towards a target population or the potential for disease emergence in emerging disease hotspots.

The maintenance and transmission function concepts can be related to the roles of vectors in vector-borne disease ecology. Blood-feeding arthropod vectors that transmit a pathogen between hosts [52] may be involved in distinct epidemiological functions, including the transmission function. The term “bridge vector” has already been used (e.g. [53,54]) to group mosquitoes that transmit West Nile Virus to humans (here the target population). However, so far, the distinction between the maintenance and transmission function has not been properly defined. This distinction could be important if maintenance and bridge vectors are different species, opening different control strategy options (i.e. on the maintenance or on the bridge hosts).

The identification of bridge hosts for a given pathogen in a given ecosystem has consequences for disease management, surveillance and control. Once bridge hosts are known, managers can adopt mitigation strategies specifically aimed at reducing contact between the target and the bridge populations. In the case of AIV, this mitigation can be achieved through strengthening biosecurity measures or decreasing the quantity of attractors on the farm (e.g. water sources or open feedlots) [23]. The adoption of adequate management measures targeting contacts between maintenance, bridge and target hosts is also more environmentally acceptable than controlling (wild) host populations.

The distinction between maintenance and bridge hosts may under some circumstances be difficult. In the case of AIV, for example, our current level of knowledge about the maintenance hosts and the apparent lack of contact in some ecosystems between the maintenance community and the target populations suggest a role for bridge hosts. The identification of hosts that do not fit into either maintenance or target host groups, as in [23], raises two possibilities: either these susceptible hosts act as bridge hosts, or they may act as previously unknown maintenance hosts for AIV epidemiology. To differentiate between these two hypotheses may require focused experimental research, for example by using infection of captive animals to determine their capacity to maintain the virus. Other approaches using meta-analysis of existing data sets have also been proposed [55]. In both cases, our conceptual framework helps with framing hypotheses based on current knowledge and using empirical tests to either confirm these hypotheses or call for a revision of our understanding of the epidemiological system (e.g. this host is not a bridge host and therefore has no (or another) role in the local context).

Our framework does have some weaknesses. In particular, proving that a bridge host in a complex multi-host system where maintenance communities are composed of numerous interacting populations does not take part in the maintenance function (i.e. that removing the bridge host will not drive the pathogen to extinction, according to Haydon et al. [10]) may necessitate an experimental design that would be difficult to achieve in practice [9]. In addition, only cases in which maintenance and target populations are not in contact have been considered so far. If they are loosely in contact (case A’ in Figure 1), the frequency and efficacy of contacts between different pairs (maintenance-target, maintenance-bridge and bridge-target) would need to be weighted against each other. Decreasing the maintenance-target contacts through management will reveal the relative importance of bridge-target contacts and could require interventions in order to efficiently stop pathogen transmission (as in the case of control of possums for bTB in New Zealand mentioned earlier). Finally, we have assumed that a bridge host must be competent for the pathogen but in some cases simple mechanical transmission (e.g., a bird carrying the virus on its feathers [56]) may be possible, relaxing the prerequisite on host competence for the bridge host.

## Conclusions

The development of complex human/livestock/wildlife interfaces, due to the encroachment of human activities within natural ecosystems, triggers new epidemiological dynamics that may permit a range of wild or domestic bridge hosts to link maintenance communities with new target hosts [57]. We would expect that domestic species and newly farmed or traded wildlife species will increasingly play bridge host roles in the emergence of new zoonoses. The epidemiology of Ebola, SARS, Lyme disease, and H1N1 AIV, for example, are not yet fully understood but are known to involve multiple hosts. We believe that introducing our definitions and operational framework into research and surveillance could contribute to more efficient use of resources to fill some knowledge gaps.

Our approach builds on that of Haydon et al. [10] and refines it to take into account potential circumstances under which an extra conceptual development is necessary. Whether this extra development will be necessary in many multi-host systems or will be used only under exceptional circumstances will be answered by studies to come. The examples given here indicate that they could be used for at least a few important diseases. The recent appearance in the epidemiological literature of similar concepts [19,23,38] that are not always placed soundly within a conceptual framework and/or ignore previous definitions suggests also the need for a consolidated review and refinement of these concepts and definitions. While no individual element of our proposed framework is new, it is clear from our discussion above that approaching the problem of understanding multi-host disease systems from a more integrated, functional perspective has the potential to offer a wide range of valuable insights into both epidemiology and its applications to pathogen control. Our approach, which requires both epidemiological and ecological approaches (and also social science approaches when the human host is considered) fits well within current initiatives that call for more transdisciplinary integration between the health sciences and other fields of research.

Finally, the global fight against emerging infectious diseases is increasingly focused on identifying potential emerging pathogens from high-risk maintenance hosts (e.g., bats and rodents, [58,59]). Recent advances in genetics and genomics have increased drastically the pace at which new micro-organisms are discovered and identified [60]. But adding new names to the list of parasites and pathogens does not provide information on which of these microorganisms might present a significant threat to animal or human health. A maintenance population hosting a large range of potentially new emerging pathogens does not constitute a threat for target populations if no transmission route exists between the maintenance and target populations. Focusing on pathogen transmission pathways, including potential hosts bridging the gap between maintenance and target populations, will help to guide “pathogen hunting” approaches as functional ecology complements taxonomy. Such an approach will help to guide high-throughput sequencing tools towards key hosts within a given epidemiological context, increasing the efficiency of surveillance and control efforts.

## Additional files

Additional file 1:
**Virus detection in African non-maintenance wild bird populations (order and family level).** This file describes the methodology that was used to gather all the relevant information on RT-PCR AIV results of non-anseriformes and non-charadriiformes species in Africa and provides the summary of findings at the bird order and family level. [24,26,31,37,61-69]. Additional file 2:
**AIV RT-PCR results for 8738 wild birds sampled in Africa until 2012.** This table displays detailed results of wild bird species and families sampled for AIV (RT-PCR) in Africa until 2012 following the gathering of data as described in Additional file 1.
