# Supplementary material for: Bridge hosts, a missing link for disease ecology in multi-host systems
Source: Vet Res. 2015 Jul 21;46(1):83. doi: 10.1186/s13567-015-0217-9 (PMC4509689; doi:10.1186/s13567-015-0217-9)
Supplement: Additional file 1: — Virus detection in African non-maintenance wild bird populations (order and family level). This file describes the methodology that was used to gather all the relevant information on RT-PCR AIV results of non-anseriformes and non-charadriiformes species in Africa and provides the summary of findings at the bird order and family level. [24,26,31,37,61-69]. [file 13567_2015_217_MOESM1_ESM.docx]

At the African continent level, we: 1) searched the literature for AIV sampling in non-anseriforms and non-charadriiforms species; 2) report on AIV sampling in non-anseriforms and non-charadriiforms species for recent surveillance and research project we implemented (original data). We compiled results already published [27,32,38,62-66,68,69] on AIV infection in non-anseriforms and non-charadriiforms bird species in sub-Saharan Africa (*n* = 3648 wild bird tested). We report additional RT-PCR results from 5090 wild birds sampled, making a total sample size of 8738 wild bird samples, including 292 species, 62 families and 19 orders in 25 countries (see these articles for the procedures of sample collection and diagnostic).

Average sample size at the species level was *n* = 29 with a standard error of 91.4 and a median of 4. We calculated the AIV infection rate for each species/family/order as the number of AIV positive samples divided by the number of bird samples. The global AIV infection rate was 0.84%. If we consider an AIV infection rate of 1% as being significant (e.g. charadriiforms have a global infection rate ranging between 0.8 and 1.4 according to families and are considered maintenance host for AIV; [25], the necessary sample size to detect at least one positive sample with a 95% confidence is *n* = 299 [67]. Interestingly, all species, family or order in our dataset with a sample size above 299 had at least one positive sample for AIV arguing for a wide host competence within the bird diversity or false positive results. This is the case for 8 out of 20 orders, 27/63 families and 43/292 species, with the exception of phalacrocoracidae. However, most sample size per species are too small to conclude if the species is competent or not (mean = 29 and median = 4).

| **Order**  **Family**  *Species (latin name)* | **Number of AIV +** | **Sample Size** | **Infection rate** |
| --- | --- | --- | --- |
| **accipitriformes** | **1** | **21** | **4.76%** |
| **accipitridae** | **1** | **21** | 4.76% |
| *Milvus migrans* | *1* | *8* | *12.50%* |
| **ciconiiformes** | **9** | **1340** | **0.67%** |
| **ardeidae** | **5** | **1264** | 0.40% |
| *Bubulcus ibis* | *5* | *927* | *0.54%* |
| **ciconiidae** | **1** | **25** | 4.00% |
| *Anastomus lamelligerus* | *1* | *23* | *4.35%* |
| **scopidae** | **0** | **2** | 0.00% |
| *Scopus umbretta* | *0* | *2* | *0.00%* |
| **threskiornithidae** | **3** | **49** | 6.12% |
| *Bostrychia hagedash* | *3* | *35* | *8.57%* |
| **columbiformes** | **8** | **934** | **0.86%** |
| **columbidae** | **7** | **928** | 0.75% |
| *Columba livia* | *2* | *186* | *1.08%* |
| *Streptopelia roseogrisea* | *2* | *97* | *2.06%* |
| *Streptopelia turtur* | *3* | *145* | *2.07%* |
| **pteroclididae** | **1** | **6** | 16.67% |
| *Pterocles exustus* | *1* | *6* | *16.67%* |
| **coraciiformes** | **3** | **217** | **1.38%** |
| **alcedinidae** | **1** | **97** | 1.03% |
| *Alcedo cristata* | *1* | *14* | *7.14%* |
| **cerylidae** | **1** | **67** | 1.49% |
| *Ceryle rudis* | *1* | *67* | *1.49%* |
| **upupidae** | **1** | **4** | 25.00% |
| *Upupa africana* | *1* | *3* | *33.33%* |
| **galliformes** | **1** | **83** | **1.20%** |
| **numididae** | **1** | **56** | 1.79% |
| *Numida meleagris* | *1* | *23* | *4.35%* |
| **gruiformes** | **11** | **1634** | **0.67%** |
| **gruidae** | **0** | **1** | 0.00% |
| *Balearica regulorum* | *0* | *1* | *0.00%* |
| **rallidae** | **11** | **1633** | 0.67% |
| *Fulica atra* | *1* | *27* | *3.70%* |
| *Fulica cristata* | *7* | *578* | *1.21%* |
| *Gallinula chloropus* | *2* | *386* | *0.52%* |
| *Porphyrio porphyrio* | *1* | *189* | *0.53%* |
| **passeriformes** | **37** | **3356** | **1.10%** |
| **alaudidae** | **1** | **31** | 3.23% |
| *Eremopterix leucotis* | *1* | *13* | *7.69%* |
| **corvidae** | **2** | **301** | 0.66% |
| *Corvus splendens* | *2* | *301* | *0.66%* |
| **hirundinidae** | **9** | **309** | 2.91% |
| *Hirundo rustica* | *8* | *208* | *3.85%* |
| *Riparia paludicola* | *1* | *75* | *1.33%* |
| **laniidae** | **1** | **6** | 16.67% |
| *Lanius collurio* | *1* | *2* | *50.00%* |
| **motacillidae** | **3** | **121** | 2.48% |
| *Anthuscinnamomeus* | *1* | *12* | *8.33%* |
| *Macronyx croceus* | *1* | *8* | *12.50%* |
| *Motacilla flava* | *1* | *25* | *4.00%* |
| **muscicapidae** | **2** | **169** | 1.18% |
| *Luscinia spp.* | *1* | *1* | *100.00%* |
| *Phoenicurus phoenicurus* | *1* | *1* | *100.00%* |
| **passeridae** | **4** | **440** | 0.91% |
| *Passer diffusus* | *1* | *2* | *50.00%* |
| *Passer domesticus* | *3* | *156* | *1.92%* |
| **phylloscopidae** | **2** | **99** | 2.02% |
| *Phylloscopus trochilus* | *2* | *3* | *66.67%* |
| **ploceidae** | **8** | **976** | 0.82% |
| *Euplectes capensis* | *1* | *2* | *50.00%* |
| *Ploceus cucullatus* | *2* | *62* | *3.23%* |
| *Ploceus velatus* | *1* | *44* | *2.27%* |
| *Quelea quelea* | *4* | *485* | *0.82%* |
| **pycnonotidae** | **1** | **122** | 0.82% |
| *Pycnonotus nigricans* | *1* | *2* | *50.00%* |
| **sylviidae** | **4** | **63** | 6.35% |
| *Bradypterus baboecala* | *1* | *5* | *20.00%* |
| *Hippolais icterina* | *1* | *1* | *100.00%* |
| *Sylvia borin* | *1* | *1* | *100.00%* |
| *Sylvia communis* | *1* | *1* | *100.00%* |
| **pelecaniformes** | **3** | **662** | **0.45%** |
| **anhingidae** | **0** | **2** | 0.00% |
| **pelicanidae** | **2** | **319** | 0.63% |
| *Pelecanus onocrotalus* | *2* | *319* | *0.63%* |
| **threskiornithidae** | **1** | **126** | 0.79% |
| *Plegadis falcinellus* | *1* | *15* | *6.67%* |

Data extracted from the Influenza Research Database [70]
