# Supplementary material for: Bridge hosts, a missing link for disease ecology in multi-host systems
Source: Vet Res. 2015 Jul 21;46(1):83. doi: 10.1186/s13567-015-0217-9 (PMC4509689; doi:10.1186/s13567-015-0217-9)
Supplement: Additional file 2: — AIV RT-PCR results for 8738 wild birds sampled in Africa until 2012. This table displays detailed results of wild bird species and families sampled for AIV (RT-PCR) in Africa until 2012 following the gathering of data as described in Additional file 1. [file 13567_2015_217_MOESM2_ESM.docx]

| **Order**  **Family**  *Species (latin name)* | **Number of AIV +** | **Sample Size** |
| --- | --- | --- |
| **accipitriformes** | **1** | **21** |
| **accipitridae** | **1** | **21** |
| *Circus aeruginosus* | *0* | *6* |
| *Circus ranivorus* | *0* | *2* |
| *Haliaeetus vocifer* | *0* | *1* |
| *Milvus migrans* | *1* | *8* |
| *nr* | *0* | *4* |
| **apodiformes** | **0** | **2** |
| **apodidae** | **0** | **2** |
| *Apus affinis* | *0* | *1* |
| *Apus caffer* | *0* | *1* |
| **bucerotiformes** | **0** | **2** |
| **upupidae** | **0** | **2** |
| *Upupa epops* | *0* | *2* |
| **caprimulgiformes** | **0** | **6** |
| **caprimulgidae** | **0** | **6** |
| *Caprimulgus climacurus* | *0* | *1* |
| *Caprimulgus fossii* | *0* | *1* |
| *Caprimulgus madagascariensis* | *0* | *1* |
| *Caprimulgus rufigena* | *0* | *2* |
| *Caprimulgus tristigma* | *0* | *1* |
| **ciconiiformes** | **9** | **1340** |
| **ardeidae** | **5** | **1264** |
| *Ardea alba melanorhynchos* | *0* | *3* |
| *Ardea cinerea* | *0* | *29* |
| *Ardea goliath* | *0* | *1* |
| *Ardea melanocephala* | *0* | *3* |
| *Ardea purpurea* | *0* | *44* |
| *Ardeola ralloides* | *0* | *91* |
| *Bubulcus ibis* | *5* | *927* |
| *Butorides striata* | *0* | *7* |
| *Casmerodius albus* | *0* | *2* |
| *Egretta ardesiaca* | *0* | *24* |
| *Egretta garzetta* | *0* | *31* |
| *Egretta intermedia* | *0* | *24* |
| *Egretta spec.* | *0* | *4* |
| *Ixobrychus minutus* | *0* | *42* |
| *Ixobrychus sturmii* | *0* | *1* |
| *nr* | *0* | *3* |
| *Nycticorax nycticorax* | *0* | *28* |
| **ciconiidae** | **1** | **25** |
| *Anastomus lamelligerus* | *1* | *23* |
| *Ciconia ciconia* | *0* | *1* |
| *Leptoptilos crumeniferus* | *0* | *1* |
| **scopidae** | **0** | **2** |
| *Scopus umbretta* | *0* | *2* |
| **threskiornithidae** | **3** | **49** |
| *Bostrychia hagedash* | *3* | *35* |
| *Platalea alba* | *0* | *1* |
| *Platalea leucorodia* | *0* | *1* |
| *Plegadis falcinellus* | *0* | *11* |
| *Threskiornis aethiopicus* | *0* | *1* |
| **coliiformes** | **0** | **33** |
| **coliidae** | **0** | **33** |
| *Colius striatus* | *0* | *24* |
| *nr* | *0* | *9* |
| **columbiformes** | **8** | **934** |
| **columbidae** | **7** | **928** |
| *Columba guinea* | *0* | *33* |
| *Columba livia* | *2* | *186* |
| *nr* | *0* | *166* |
| *Oena capensis* | *0* | *20* |
| *Streptopelia capicola* | *0* | *78* |
| *Streptopelia decaocto* | *0* | *1* |
| *Streptopelia decipiens* | *0* | *5* |
| *Streptopelia hypopyrrha* | *0* | *64* |
| *Streptopelia roseogrisea* | *2* | *97* |
| *Streptopelia semitorquata* | *0* | *18* |
| *Streptopelia senegalensis* | *0* | *90* |
| *Streptopelia turtur* | *3* | *145* |
| *Streptopelia vinacea* | *0* | *5* |
| *Turtur abyssinicus* | *0* | *17* |
| *Turtur afer* | *0* | *3* |
| **pteroclididae** | **1** | **6** |
| *Pterocles exustus* | *1* | *6* |
| **coraciiformes** | **3** | **217** |
| **alcedinidae** | **1** | **97** |
| *Alcedo atthis* | *0* | *28* |
| *Alcedo cristata* | *1* | *14* |
| *Ceryle rudis* | *0* | *20* |
| *Halcyon leucocephala* | *0* | *3* |
| *Halcyon smyrnensis* | *0* | *11* |
| *nr* | *0* | *21* |
| **bucerotes** | **0** | **2** |
| *Promerops purpureus* | *0* | *2* |
| **cerylidae** | **1** | **67** |
| *Ceryle rudis* | *1* | *67* |
| **coraciidae** | **0** | **3** |
| *Coracias caudatus* | *0* | *3* |
| *Eurystomus glaucurus* | *0* | *1* |
| *nr* | *0* | *1* |
| **dacelonidae** | **0** | **8** |
| *Halcyon albiventris* | *0* | *6* |
| *Halcyon leucocephala* | *0* | *1* |
| *Halcyon senegalensis* | *0* | *1* |
| **meropidae** | **0** | **36** |
| *Merops albicolis* | *0* | *5* |
| *Merops apiaster* | *0* | *1* |
| *Merops bulocki* | *0* | *18* |
| *Merops persicus* | *0* | *12* |
| **upupidae** | **1** | **4** |
| *Upupa africana* | *1* | *3* |
| *Upupa epops* | *0* | *1* |
| **culculiformes** | **0** | **14** |
| **cuculidae** | **0** | **14** |
| *Centropus senegalensis* | *0* | *5* |
| *Centropus superciliosus* | *0* | *2* |
| *Centropus toulou* | *0* | *4* |
| *Clamator glandarius* | *0* | *1* |
| *nr* | *0* | *2* |
| **falconiformes** | **0** | **6** |
| **accipitridae** | **0** | **2** |
| *Circus macrourus* | *0* | *1* |
| *Elanus caeruleus* | *0* | *1* |
| **falconidae** | **0** | **4** |
| *Falco chicquera* | *0* | *1* |
| *Falco columbarius* | *0* | *1* |
| *Falco peregrinus* | *0* | *1* |
| *Falco tinnunculus* | *0* | *1* |
| **galliformes** | **1** | **83** |
| **numididae** | **1** | **56** |
| *Meleagris gallopavo* | *0* | *33* |
| *Numida meleagris* | *1* | *23* |
| **phasianidae** | **0** | **27** |
| *Coturnix coturnix* | *0* | *4* |
| *Francolinus bicalcaratus* | *0* | *2* |
| *Pternistis adspersus* | *0* | *8* |
| *Pternistis capensis* | *0* | *7* |
| *Pternistis swainsonii* | *0* | *5* |
| *Ptilopachus petrosus* | *0* | *1* |
| **gruiformes** | **11** | **1634** |
| **gruidae** | **0** | **1** |
| *Balearica regulorum* | *0* | *1* |
| **rallidae** | **11** | **1633** |
| *Amaurornis flavirostris* | *0* | *96* |
| *Dryolimnas cuvieri* | *0* | *28* |
| *Fulica atra* | *1* | *27* |
| *Fulica cristata* | *7* | *578* |
| *Gallinula angulata* | *0* | *5* |
| *Gallinula chloropus* | *2* | *386* |
| *nr* | *0* | *1* |
| *Porphyrio alleni* | *0* | *262* |
| *Porphyrio madagascariensis* | *0* | *5* |
| *Porphyrio porphyrio* | *1* | *189* |
| *Porzana porzana* | *0* | *3* |
| *Rallus caerulescens* | *0* | *27* |
| *Rallus madagascariensis* | *0* | *26* |
| **passeriformes** | **37** | **3356** |
| **acrocephaildae** | **0** | **50** |
| *Acrocephalus gracilirostris* | *0* | *2* |
| *Acrocephalus palustris* | *0* | *1* |
| *Acrocephalus schoenobaenus* | *0* | *26* |
| *Acrocephalus scirpaceus* | *0* | *10* |
| *Acrocephalus stentoreus* | *0* | *11* |
| **alaudidae** | **1** | **31** |
| *Calandrella cinerea* | *0* | *8* |
| *Eremopterix leucotis* | *1* | *13* |
| *Galerida cristata* | *0* | *5* |
| *Galerida modesta* | *0* | *1* |
| *Mirafra africana* | *0* | *3* |
| *nr* | *0* | *1* |
| **campephagidae** | **0** | **1** |
| *Campephaga phoenicea* | *0* | *1* |
| **cisticolidae** | **0** | **48** |
| *Apalis flavida* | *0* | *2* |
| *Camaroptera brachyura* | *0* | *4* |
| *Camaroptera brevicaudata* | *0* | *12* |
| *Cisticola chiniana* | *0* | *2* |
| *Cisticola emini* | *0* | *2* |
| *Cisticola galactotes* | *0* | *10* |
| *Cisticola juncidis* | *0* | *1* |
| *Cisticola natalensis* | *0* | *1* |
| *Cisticola tinniens* | *0* | *1* |
| *Hypergerus atriceps* | *0* | *5* |
| *Prinia gracilis* | *0* | *8* |
| **corvidae** | **2** | **301** |
| *Corvus splendens* | *2* | *301* |
| **dicruridae** | **0** | **1** |
| *nr* | *0* | *1* |
| **emberizidae** | **0** | **2** |
| *Emberiza tahapisi* | *0* | *1* |
| *nr* | *0* | *1* |
| **estrildidae** | **0** | **190** |
| *Amandava amandava* | *0* | *18* |
| *Estrilda astrild* | *0* | *2* |
| *Estrilda caerulescens* | *0* | *16* |
| *Estrilda melpoda* | *0* | *11* |
| *Estrilda troglodytes* | *0* | *4* |
| *Lagonosticta rara* | *0* | *1* |
| *Lagonosticta rufopicta* | *0* | *14* |
| *Lagonosticta sanguinodorsalis* | *0* | *20* |
| *Lagonosticta senegala* | *0* | *13* |
| *Lonchura cucullata* | *0* | *2* |
| *Lonchura malabarica* | *0* | *18* |
| *Nesocharis capistrata* | *0* | *1* |
| *nr* | *0* | *52* |
| *Ortygospiza atricollis* | *0* | *2* |
| *Pytilia melba* | *0* | *1* |
| *Spermestes cucullatus* | *0* | *1* |
| *Uraeginthus angolensis* | *0* | *1* |
| *Uraeginthus bengalus* | *0* | *13* |
| **fringillidae** | **0** | **37** |
| *nr* | *0* | *37* |
| **hirundinidae** | **9** | **309** |
| *Hirundo albigularis* | *0* | *2* |
| *Hirundo fuligula* | *0* | *1* |
| *Hirundo rustica* | *8* | *208* |
| *Hirundo smithii* | *0* | *1* |
| *nr* | *0* | *9* |
| *Riparia paludicola* | *1* | *75* |
| *Riparia paludicola ducis* | *0* | *5* |
| *Riparia riparia* | *0* | *8* |
| **Indicatoridae** | **0** | **1** |
| *Indicator indicator* | *0* | *1* |
| **laniidae** | **1** | **6** |
| *Lanius collaris* | *0* | *2* |
| *Lanius collurio* | *1* | *2* |
| *Lanius excubitor* | *0* | *2* |
| **malaconotidae** | **0** | **34** |
| *Dryoscopus gambensis* | *0* | *1* |
| *Laniarius aethiopicus* | *0* | *2* |
| *Laniarius barbarus* | *0* | *10* |
| *nr* | *0* | *7* |
| *Tchagra senegalus* | *0* | *8* |
| *Telophorus sulfureopectus* | *0* | *6* |
| **megaluridae** | **0** | **2** |
| *Locustella fluviatilis* | *0* | *1* |
| *Locustella luscinioides* | *0* | *1* |
| **monarchidae** | **0** | **6** |
| *nr* | *0* | *2* |
| *Terpsiphone viridis* | *0* | *4* |
| **motacillidae** | **3** | **121** |
| *Anthus campestris* | *0* | *5* |
| *Anthus cervinus* | *0* | *1* |
| *Anthus cinnamomeus* | *0* | *1* |
| *Anthus spinoletta* | *0* | *3* |
| *Anthus trivialis* | *0* | *2* |
| *Anthuscinnamomeus* | *1* | *12* |
| *Macronyx ameliae* | *0* | *10* |
| *Macronyx capensis* | *0* | *9* |
| *Macronyx croceus* | *1* | *8* |
| *Motacilla aguimp* | *0* | *1* |
| *Motacilla alba* | *0* | *21* |
| *Motacilla capensis* | *0* | *8* |
| *Motacilla flava* | *1* | *25* |
| *nr* | *0* | *15* |
| **muscicapidae** | **2** | **169** |
| *Bradornis mariquensis* | *0* | *1* |
| *Bradornis pallidus* | *0* | *2* |
| *Cercomela familiaris* | *0* | *21* |
| *Cossypha albicapilla albicapilla* | *0* | *4* |
| *Cossypha caffra* | *0* | *1* |
| *Cossypha niveicapilla* | *0* | *20* |
| *Cossypha polioptera* | *0* | *1* |
| *Erithacus rubecula* | *0* | *4* |
| *Luscinia spp.* | *1* | *1* |
| *Luscinia svecica* | *0* | *90* |
| *Melaenornis edolioides* | *0* | *4* |
| *Myrmecocichla aethiops* | *0* | *2* |
| *Myrmecocichla albifrons* | *0* | *1* |
| *nr* | *0* | *3* |
| *Phoenicurus phoenicurus* | *1* | *1* |
| *Saxicola rubetra* | *0* | *1* |
| *Saxicola torquatus* | *0* | *5* |
| *Thamnolaea cinnamomeiventris* | *0* | *7* |
| **nectariniidae** | **0** | **29** |
| *Nectarinia jugularis* | *0* | *4* |
| *Nectarinia olivacea* | *0* | *4* |
| *Nectarinia senegalensis* | *0* | *7* |
| *Nectarinia venusta* | *0* | *2* |
| *Nectarinia verticalis* | *0* | *4* |
| *nr* | *0* | *8* |
| **passeridae** | **4** | **440** |
| *nr* | *0* | *58* |
| *Passer diffusus* | *1* | *2* |
| *Passer domesticus* | *3* | *156* |
| *Passer griseus* | *0* | *7* |
| *Passer hispaniolensis* | *0* | *38* |
| *Passer luteus* | *0* | *167* |
| *Passer melanurus* | *0* | *4* |
| *Passer motitensis* | *0* | *1* |
| *Petronia dentata* | *0* | *2* |
| *Plocepasser superciliosus* | *0* | *5* |
| **phylloscopidae** | **2** | **99** |
| *Phylloscopus collybita* | *0* | *95* |
| *Phylloscopus sibilatrix* | *0* | *1* |
| *Phylloscopus trochilus* | *2* | *3* |
| **platysteiridae** | **0** | **14** |
| *Batis senegalensis* | *0* | *5* |
| *nr* | *0* | *1* |
| *Platysteira cyanea* | *0* | *8* |
| **ploceidae** | **8** | **976** |
| *Anaplectes melanotis* | *0* | *1* |
| *Euplectes afer* | *0* | *2* |
| *Euplectes albonotatus* | *0* | *2* |
| *Euplectes axillaris* | *0* | *14* |
| *Euplectes capensis* | *1* | *2* |
| *Euplectes franciscanus* | *0* | *37* |
| *Euplectes hordeaceus* | *0* | *26* |
| *Euplectes orix* | *0* | *71* |
| *Euplectes progne* | *0* | *1* |
| *Foudia madagascariensis* | *0* | *16* |
| *nr* | *0* | *135* |
| *Ploceus cucullatus* | *2* | *62* |
| *Ploceus heuglini* | *0* | *10* |
| *Ploceus intermedius* | *0* | *3* |
| *Ploceus luteolus* | *0* | *3* |
| *Ploceus nigricollis* | *0* | *27* |
| *Ploceus ocularis* | *0* | *1* |
| *Ploceus sp.* | *0* | *1* |
| *Ploceus velatus* | *1* | *44* |
| *Ploceus vitellinus* | *0* | *18* |
| *Quelea quelea* | *4* | *485* |
| *Sporopipes frontalis* | *0* | *15* |
| **pycnonotidae** | **1** | **122** |
| *Andropadus virens* | *0* | *1* |
| *Chlorocichla flavicollis* | *0* | *7* |
| *Chlorocichla simplex* | *0* | *1* |
| *nr* | *0* | *3* |
| *Pycnonotus barbatus* | *0* | *102* |
| *Pycnonotus capensis* | *0* | *3* |
| *Pycnonotus nigricans* | *1* | *2* |
| *Pycnonotus tricolor* | *0* | *3* |
| **stenostiridae** | **0** | **2** |
| *Elminia longicauda* | *0* | *2* |
| **sturnidae** | **0** | **37** |
| *Cinnyricinclus leucogaster* | *0* | *2* |
| *Creatophora cinerea* | *0* | *8* |
| *Lamprotornis chalybaeus* | *0* | *5* |
| *Lamprotornis chloropterus* | *0* | *1* |
| *Lamprotornis purpureus* | *0* | *1* |
| *Sturnus vulgaris* | *0* | *20* |
| **sylviidae** | **4** | **63** |
| *Acrocephalus schoenobaenus* | *0* | *1* |
| *Acrocephalus stentoreus* | *0* | *2* |
| *Bradypterus baboecala* | *1* | *5* |
| *Camaroptera brachyura* | *0* | *1* |
| *Cisticola sp.* | *0* | *6* |
| *Hippolais icterina* | *1* | *1* |
| *Melocichla mentalis* | *0* | *1* |
| *nr* | *0* | *36* |
| *Phylloscopus collybita* | *0* | *2* |
| *Prinia gracilis* | *0* | *1* |
| *Sylvia borin* | *1* | *1* |
| *Sylvia communis* | *1* | *1* |
| *Sylvia hortensis* | *0* | *1* |
| *Sylvietta brachyura* | *0* | *1* |
| *Sylvietta rufescens* | *0* | *3* |
| **timaliidae** | **0** | **200** |
| *Turdoides jardineii* | *0* | *2* |
| *Turdoides plebejus* | *0* | *11* |
| *Turdoides reinwardtii* | *0* | *187* |
| **turdidae** | **0** | **13** |
| *Luscinia svecica* | *0* | *3* |
| *nr* | *0* | *5* |
| *Saxicola caprata* | *0* | *2* |
| *Turdus libonyanus* | *0* | *1* |
| *Turdus philomelos* | *0* | *1* |
| *Turdus smithi* | *0* | *1* |
| **viduidae** | **0** | **28** |
| *Anomalospiza imberbis* | *0* | *1* |
| *nr* | *0* | *22* |
| *Vidua chalybeata* | *0* | *4* |
| *Vidua macroura* | *0* | *1* |
| **zosteropidae** | **0** | **24** |
| *nr* | *0* | *4* |
| *Zosterops capensis* | *0* | *1* |
| *Zosterops senegalensis* | *0* | *19* |
| **pelecaniformes** | **3** | **662** |
| **Anhingidae** | **0** | **2** |
| *Anhiga rufa* | *0* | *2* |
| **pelicanidae** | **2** | **319** |
| *Pelecanus onocrotalus* | *2* | *319* |
| **phalacrocoracidae** | **0** | **215** |
| *Phalacrocorax africanus* | *0* | *59* |
| *Phalacrocorax capensis* | *0* | *1* |
| *Phalacrocorax carbo* | *0* | *189* |
| *Phalacrocorax lucidus* | *0* | *5* |
| **threskiornithidae** | **1** | **126** |
| *Plegadis falcinellus* | *1* | *15* |
| *Threskiornis aethiopicus* | *0* | *111* |
| **phoenicopteriformes** | **0** | **19** |
| **phoenicopteridae** | **0** | **19** |
| *Phoenicopterus minor* | *0* | *18* |
| *Phoenicopterus roseus* | *0* | *1* |
| **piciformes** | **0** | **79** |
| **Indicatoridae** | **0** | **22** |
| *Indicator indicator* | *0* | *17* |
| *Indicator minor* | *0* | *1* |
| *Indicator willcocksi* | *0* | *1* |
| *nr* | *0* | *3* |
| **lybiidae** | **0** | **52** |
| *Lybius dubius* | *0* | *3* |
| *Lybius leucocephalus* | *0* | *2* |
| *Lybius torquatus* | *0* | *2* |
| *Lybius veilloti* | *0* | *5* |
| *nr* | *0* | *16* |
| *Pogoniulus chrysoconus* | *0* | *21* |
| *Pogoniulus scolopaceus* | *0* | *2* |
| *Trachyphonus vaillantii* | *0* | *1* |
| **picidae** | **0** | **5** |
| *Campethera punctuligera* | *0* | *1* |
| *Dendropicos goertae* | *0* | *3* |
| *Jynx torquilla* | *0* | *1* |
| **podicipediformes** | **0** | **8** |
| **podicipedidae** | **0** | **8** |
| *Tachybaptus ruficollis* | *0* | *8* |
| **pteroclidiformes** | **0** | **58** |
| **pteroclididae** | **0** | **58** |
| *Pterocles quadricinctus* | *0* | *58* |
| **strigiformes** | **0** | **19** |
| **strigidae** | **0** | **5** |
| *Asio madagascariensis* | *0* | *4* |
| *Athene noctua* | *0* | *1* |
| **tytonidae** | **0** | **14** |
| *Tyto alba* | *0* | *14* |
